# Supplementary material for: Alpha-T-catenin is expressed in peripheral nerves as a constituent of Schwann cell adherens junctions
Source: Biol Open. 2022 Dec 19;11(12):bio059634. doi: 10.1242/bio.059634 (PMC9793867; doi:10.1242/bio.059634)
Supplement: Supplementary information [file biolopen-11-059634-s1.pdf]

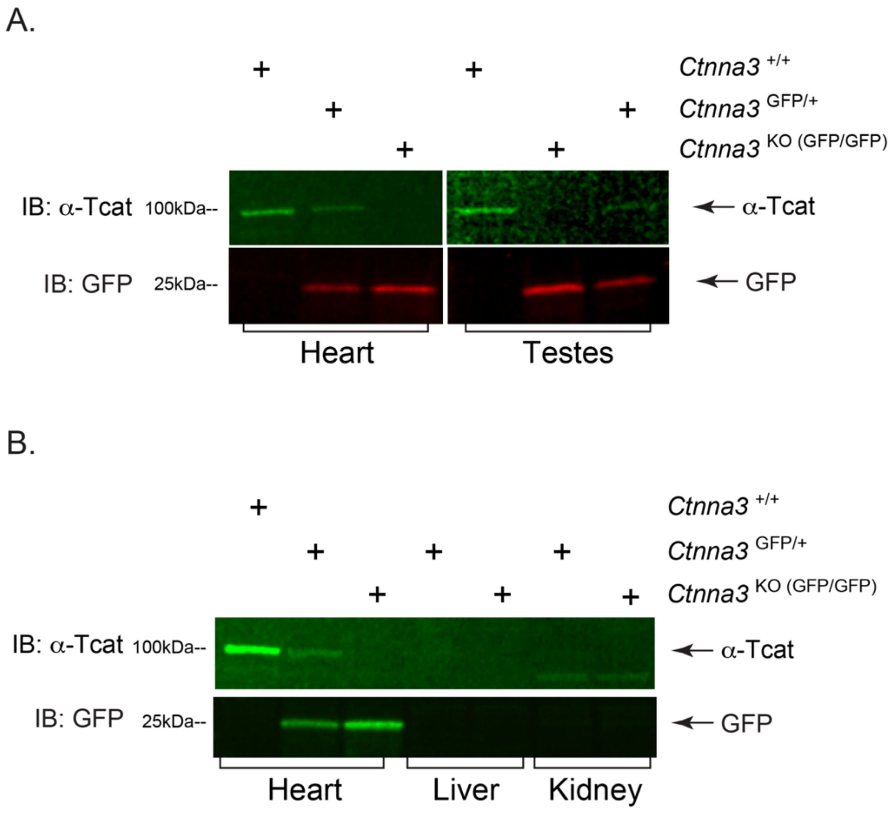

Fig. S1. Validation of *Ctnna3*^{eGFPcaax} -reporter mouse GFP expression in mouse tissues. **A.** Immunoblotting of *Ctnna3*^{WT}, *Ctnna3*$^{GFP/+}$ and *Ctnna3*$^{KO\ (GFP/GFP)}$ lysates from heart (left) and testes (right) with anti-αTcat (green) and anti-GFP (red) antibodies. Note copy- dependent increase in GFP abundance between *Ctnna3*$^{GFP/+}$ and *Ctnna3*$^{KO\ (GFP/GFP)}$ mice, with complementary reduction in αTcat protein. **B**. Immunoblotting of *Ctnna3*^{WT}, *Ctnna3*$^{GFP/+}$ and *Ctnna3*$^{KO\ (GFP/GFP)}$ lysates from heart, liver and kidney with anti-αTcat (green) and anti-GFP (red) antibodies. Note liver and kidney, which lack *Ctnna3* expression, contain no GFP protein.

Biology Open • Supplementary information

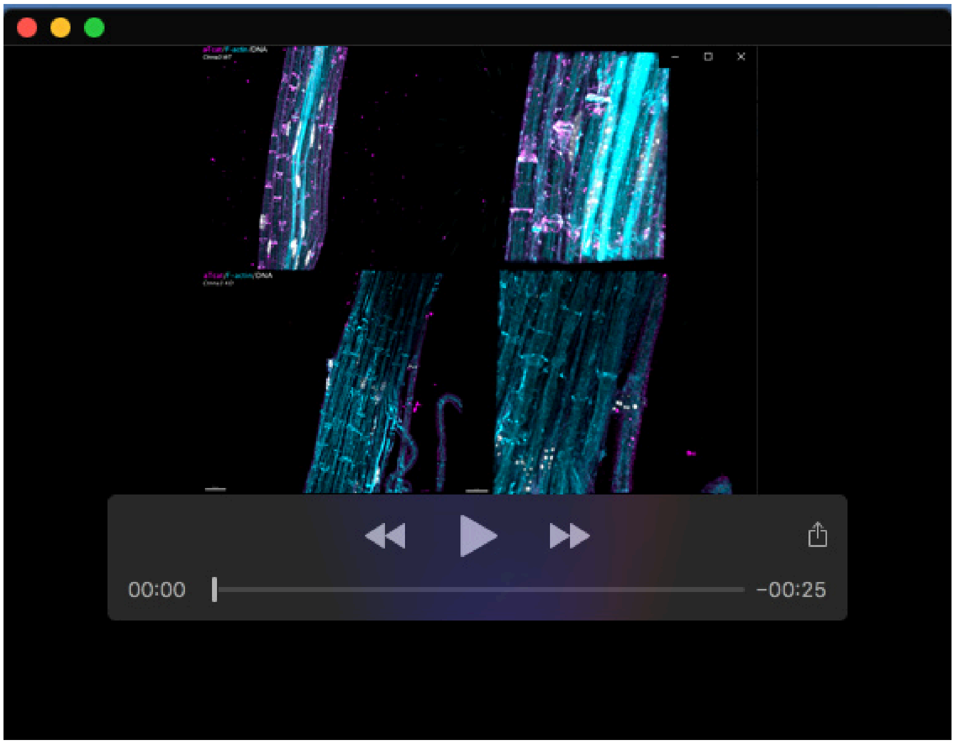

Movie 1. αTcat is expressed at myelin incisures in sciatic nerve.

Low- and high-magnification Z-stacks (0.25μm steps; 10μm depth) of sciatic nerve sections from *Ctnna3*^{WT} and Ctnna3$^{KO\ (GFP/GFP)}$ were reconstructed in 3D using Imaris. Immunofluorescence staining of myelin incisures by αTcat (magenta) and F-actin (cyan). DNA labeling by Hoechst (gray).

KEY RESOURCES

REAGENT or RESOURCE	SOURCE	IDENTIFIER
Antibodies		
Anti-alpha-T-catenin (rat monoclonal) *(immunofluorescence)*	VanRoy lab VIB; U. Ghent, BELGIUM *CONTACT: Jolanda.VanHengel@UGent.be*	1159_12A4S4; Recognizes human/mouse; Raised against MLAPKEDRLNANKNI; Goossens et al., 2007
Anti-alpha-T-catenin (rabbit polyclonal) *(immunoblotting)*	VanRoy lab VIB; U. Ghent, BELGIUM *CONTACT: Jolanda.VanHengel@UGent.be*	Anti-peptide Ab #942; Raised against KIHPLQVMSEFRGRQIY of human αT-cat Janssens et al., 2001
Anti-E-cadherin	BD Biosciences	Cat# 610182
Anti-GAPDH	EMD-Millipore	Cat# CB1001
Anti-GAPDH	Santa Cruz	Cat# SC-25778
Anti-GFP	Invitrogen	Cat# A11122
Anti-alpha-E-catenin	Enzo	Cat# ALX-804-101
Alexa Fluor 680 Phalloidin	Invitrogen	Cat# A22286
Goat anti-rabbit Alexa Fluor 568	Invitrogen	Cat# A11001
Goat anti-rat Alexa Fluor 594	Invitrogen	Cat# A11007
Goat anti-mouse Alexa Fluor 488	Invitrogen	Cat# A28175
Donkey anti-mouse IRDye 680RD or 800CW	Li-Cor	Cat# 926-68073, 926-32213
Donkey anti-rabbit IRDye 680RD or 800CW	Li-Cor	Cat# 926-68072, 926-32212

Biology Open • Supplementary information

Goat anti-rabbit IgG (H+L)-HRP	Bio-Rad	Cat# 1706515
Goat anti-rat IgG-HRP	Millipore	Cat# AP136P
Kits		
RNAeasy Plus Mini RNA isolation kit	Qiagen	Cat# 74134
Criterion 4-20% TGX acrylamide gel	Bio-Rad	Cat# 5671093 or 5671094
Transblot Turbo Transfer Pack Midi 0.2um nitrocellulose	Bio-Rad	Cat# 1704159
Protein Assay Dye Concentrate	Bio-Rad	Cat# 5000006
ECL2 Western Blot Substrate	Pierce (Thermo)	Cat# 80196
Chemicals, Peptides, and Recombinant Proteins		
Antigen retrieval buffer	Vector	Cat# H-3300
Bovine Serum Albumin (BSA) Fraction V	Calbiochem	Cat# 2930
Complete EDTA-free protease inhibitor	Millipore-Sigma (Roche)	Cat# 11836170001
Glycerol	Calbiochem	Cat# 4750
Glycine	JT Baker	Cat# 4059-02
Hoechst 33342	ThermoFisher Scientific	Cat# 62249
Intercept TBS Blocking Buffer	Li-Cor	Cat# 927-60001
Milk/Blocking solution	Bio-Rad	Cat# 170-6404

Biology Open • Supplementary information

Normal Goat Serum (NGS)	Novex (Life Technologies)	Cat# PCN5000
Paraformaldehyde (16%)	Electron Microscopy Sciences	Cat# 15710-S
Phosphate Buffered Saline (PBS)	Sigma	Cat# D5652
Poly-L-lysine	Millipore-Sigma	Cat# A005C
Ponceau S	Sigma	Cat# P3504
ProLong Gold Antifade Mountant	ThermoFisher Scientific	Cat# P36934
Tris Buffered Saline (TBS)	Bio-Rad	Cat# 1706435
Tris-Glycine-SDS Running Buffer	Bio-Rad	Cat# 1610732
Triton-X100	Sigma	Cat# T8787
Tween-20	Fisher	Cat# BP337-100
Xylene	Fisher	Cat# X3P
Zamboni's fixative	Newcomer Supply	Cat# 1459A
Mouse *Ctnna3* primers	IDT	Custom: Forward Primer (FP) 5'-GGTTACTACCCTGGTGAATTGTCC-3', Reverse Primer (RP) 5'-CTCTTTTCGAACTTCCTGGAGTGC-3'.
Experimental Models: Organisms/Strains		
Mouse: *C57BL/6-Ctnna3em1Cgot*	NU Transgenic & Targeted Mutagenesis Core Facility	Genotyping via Transnetyx (real-time qPCR validation); Jax Stock No. 037394 in process
Mouse: C57BL/6J	Jackson Labs	JAX: 000664

Biology Open • Supplementary information

Software and Algorithms		
FIJI/ImageJ (version: 2.1.0/1.53c)	Schneider, CA et al., 2012	https://imagej.nih.gov/ij/
Imaris		https://imaris.oxinst.com/
Other		
Beadblaster 24	Benchmark	D2400
Beadbug Homogenizer Tubes	Sigma	Cat# Z763802
Sonifier 450	Branson	450
MyiQ Single Color Real-Time PCR Detection System	BioRad	Cat# 170-9770
Tissue Tearor	Biospec	985370-395
Li-Cor Odyssey FC Imager	Li-Cor	
Transblot Turbo	Bio-Rad	
MZ FL III Fluorescence stereomicroscope	Leica	
W1 Spinning Disk Confocal	Nikon Instruments	
Axioplan2 epifluorescence microscope	Zeiss; 20x objective (Air)	AxioCAM HR Camera with AxioVision 4.8 software

Biology Open • Supplementary information
